# Abnormal neural oscillations during gait and dual-task in Parkinson’s disease

**DOI:** 10.3389/fnsys.2022.995375

**Published:** 2022-09-15

**Authors:** Rachel O. Nwogo, Stefan Kammermeier, Arun Singh

**Affiliations:** ^1^Division of Basic Biomedical Sciences, Sanford School of Medicine, University of South Dakota, Vermillion, SD, United States; ^2^Department of Neurology, Ludwig Maximilian University, Munich, Germany

**Keywords:** Parkinson’s disease, gait, dual-task, walking, oscillations, frequency bands, EEG

## Abstract

Gait dysfunctions are debilitating motor symptoms of Parkinson’s disease (PD) and may result in frequent falling with health complications. The contribution of the motor-cognitive network to gait disturbance can be studied more thoroughly by challenging motor-cognitive dual-task gait performances. Gait is a complex motor task that requires an appropriate contribution from motor and cognitive networks, reflected in frequency modulations among several cortical and subcortical networks. Electrophysiological recordings by scalp electroencephalography and implanted deep brain stimulation (DBS) electrodes have unveiled modulations of specific oscillatory patterns in the cortical-subcortical circuits in PD. In this review, we summarize oscillatory contributions of the cortical, basal ganglia, mesencephalic locomotor, and cerebellar regions during gait and dual-task activities in PD. We detail the involvement of the cognitive network in dual-task settings and compare how abnormal oscillations in the specific frequency bands in the cortical and subcortical regions correlate with gait deficits in PD, particularly freezing of gait (FOG). We suggest that altered neural oscillations in different frequencies can cause derangements in broader brain networks, so neuromodulation and pharmacological therapies should be considered to normalize those network oscillations to improve challenged gait and dual-task motor functions in PD. Specifically, the theta and beta bands in premotor cortical areas, subthalamic nucleus, as well as alpha band activity in the brainstem prepontine nucleus, modulate under clinically effective levodopa and DBS therapies, improving gait and dual-task performance in PD with FOG, compared to PD without FOG and age-matched healthy control groups.

## Introduction

The defining motor symptoms in idiopathic Parkinson’s disease (PD) are bradykinesia and rigidity with specific postural and gait abnormalities (resting tremor is an optional and accessory symptom) (Pringsheim et al., 2021). Progress of severity greatly reduces quality of life over the course of one to two decades, which results in immobility and high mortality predominantly from pulmonary infectious complications (Iwasaki et al., 1990). Freezing of gait (FOG) is a common disabling gait dysfunction defined as a “brief, episodic absence or significant reduction of forward movement of the feet despite the intention to walk” (Vercruysse et al., 2012), with an optional preceding increase of step cadence with shortened step length, asymmetric mobility of the legs, the plantar surface or toes remaining in contact with the surface, and/or alternating leg trembling at 3–8 Hz (Nutt et al., 2011; Nonnekes et al., 2019). Mostly, FOG appears around a median of 6 years after disease onset (Prasad et al., 2018), splitting the PD population into early- versus late-onset around the 6-year mark. Due to these characteristics, FOG defines an independent risk factor for falls with a positive predictive value of 0.75 and a negative predictive value of 0.73 (Latt et al., 2009).

**FIGURE 1:**
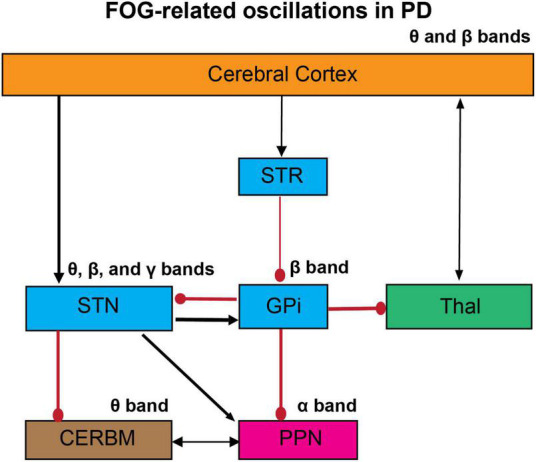
The proposed oscillatory mechanism of FOG in PD. The abnormal gait in PD can be mediated by aberrant changes in the oscillatory activities across cortical and subcortical networks. FOG is associated with impaired cortico-striatal-thalamic circuits, which lead to an increase in glutamatergic input in the STN and pallidal inhibitory outflow. These abnormalities have been examined by the changes in neural and neuronal levels in humans and animal models. Here we propose the abnormalities in oscillatory activities in the whole networks, studied by EEG and LFP recordings. These studies suggest that the abnormal oscillations in these circuits can induce impaired co-ordination of flexor-extensor pairs in the lower limbs and ultimately the FOG. STR, striatum; GPi, globus pallidus internus; STN, subthalamic nucleus; Thal, thalamus; CERBM, cerebellum; PPN, pedunculopontine nucleus; Black arrows, excitatory; Red circles, inhibitory. The thickness of the arrows resembles to their presumed activity.

FOG in PD is a complex phenomenon, whose underlying mechanisms have not been entirely understood. Neuronal connectivity of FOG has been discussed previously (Lewis and Shine, 2016). However, the neural oscillatory patterns involved have not been reviewed thoroughly yet. The basal ganglia model has suggested that its output structures, namely the globus pallidus internus (GPi) and substantia nigra pars reticulata (SNr), provide the largest inhibitory afferent connections to thalamic and brainstem regions (Nambu, 2004). This implies that aberrant activity in these nuclei may cause the freezing phenomenon in PD (Lewis and Shine, 2016). Severe dopamine loss is associated with hypoactivity in the striatum, resulting in increased inhibitory output from the basal ganglia circuits and, therefore, may be responsible for freezing episodes in the advanced stages of PD (Shine et al., 2013a). Additionally, projections from the prefrontal and supplementary motor cortical regions emit excitatory glutamatergic efferents to the striatal region. Therefore, abnormal activity within these cortical regions may cause atypical activity in the striatum, consecutively affecting the downstream structures GPi and SNr. Another mechanism of FOG can be observed by examining the subthalamic nucleus (STN) networks. The STN sends forth pronounced excitatory efferents into GPi/SNr. An increased firing rate in the STN further augments the firing rate within GPi/SNr, which in turn decreases the activity in the PPN; therefore STN can also induce freezing episodes in PD (Shine et al., 2013b). Here, we reviewed studies of cortical and subcortical oscillatory mechanism involvement in gait dysfunction and FOG in PD (Figure 1). These studies have shown the occurrence and propagation of oscillations in the different frequency bands among complex oscillatory networks on cortical and subcortical levels, which may cause the freezing phenomenon in PD.

There is a probabilistic presentation of FOG episodes in experimental human trials, even with identical environmental conditions, pharmacological steady state, and within the same patient. FOG may not be exhibited in one experimental iteration, but in the next one. Given the experimental difficulty to evaluate FOG, researchers are developing more sensitive and specific clinical and neurophysiological approaches. The occurrence of FOG exhibits high variability and can depend on environmental triggers, emotional state, cognitive loading, and medication; therefore, the frequency and the time of FOG episodes are difficult to measure across PD populations. Moreover, there are methodological limitations to study FOG in the laboratory environment because FOG is a stochastic process and patients who are normally confronted with it on a daily basis are frequently not able to produce it when tested in a gait laboratory. This may be a result of the physical environment, such as visual cues or increased awareness in the testing situation, reducing the likelihood of FOG occurrence.

Recent evidence has emphasized the role of cognitive control in lower-extremity movement and gait with the use of dual-task experiments in combined cognitive and motor tasks in PD subjects (Morris et al., 2016; Singh et al., 2018; Walton et al., 2018). Depending on the level of impairment, dual-task experiments are designed to challenge motor control systems into displaying a wider range of dysfunction than single-task challenges alone (Springer et al., 2006; Longo et al., 2018). Since daily motor activities are regularly performed simultaneously with other tasks, and most PD-related falls occur “while doing another task with cognitive loading,” gait experiments should be conducted particularly in these conditions to yield clinically relevant results. During dual-task conditions, PD patients are characterized by higher gait asymmetry, decreased bilateral coordination, and higher gait variability compared to age-matched healthy controls (Hausdorff et al., 2003; Plotnik et al., 2007, 2011a,2011b; Yogev-Seligmann et al., 2012).

**FIGURE 2:**
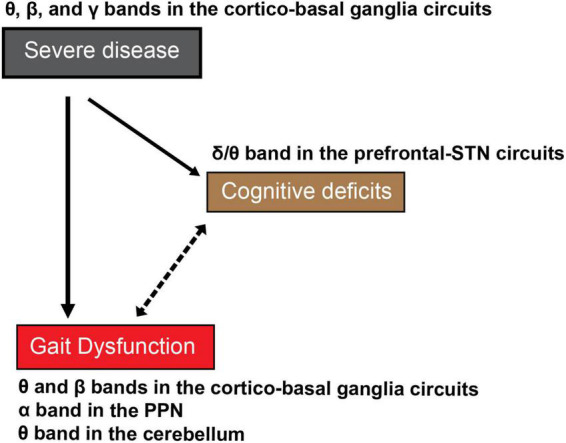
The proposed cognitive deficits-related oscillations in the cortical and subcortical networks in gait dysfunction in PD. As disease severity increases with time, patients with PD exhibit cognitive deficits, as well as severe gait dysfunction. The indirect association between severe gait dysfunctions such as FOG and cognitive deficits in the advanced stage of PD remains undetermined. The reciprocal effects of disease severity and cognitive deficits-related oscillations have been proposed as the mechanism of severe gait dysfunction or FOG in PD. Continous lines indicate definite causal relation and the dotted line indicates associative mutual relationship without proven causality.

As the disease progresses, PD patients also exhibit more expressed deficits during dual-task activities compared to single-task testing, since a more pronounced cognitive impairment can be seen in the advanced stages of the disease (Kelly et al., 2012a,b; Stegemoller et al., 2014; Scholl et al., 2021). Recent studies have demonstrated the contribution of cortical and subcortical neural activity to single- and dual-task lower-extremity motor and gait performances (Singh et al., 2013, 2020; Pizzamiglio et al., 2017; Singh, 2018). Since gait symptoms are exacerbated by cognitive loading, and oscillations in certain neural networks are correlated with motor-cognitive dual-task performance, their mutual correlation and possible causation must be considered (Figure 2).

The use of dual- or multiple tasks to define or enhance neural processing networks has not come without theoretical criticism, particularly for double cognitive tasks (Hommel, 2020; Esmaeili Bijarsari, 2021). For cognitive-motor interference, the theories of processing bottleneck, capacity-sharing, and/or crosstalk serve as empirical evidence, even though a definite histo-anatomical correlate in the brain is still missing (Leone et al., 2017). The mutual interconnectivity of the basal ganglia receiving, cerebellar-receiving, and associative cortical regions of the thalamic subnuclei has been proposed as a strong candidate (Prevosto and Sommer, 2013; Cover and Mathur, 2021).

Overall, our review examines cortical and subcortical oscillatory activity in PD patients involved in cognitive-motor dual-task activities.

### Contribution of cognitive domains in gait abnormalities

Contributing factors to PD gait disturbance are level of disease progression (usually measured by the Unified Parkinson’s Disease Rating Scale (UPDRS), part III: motor rating scale), basophobia (fear of falling), musculoskeletal ailments and physical inactivity, proprioceptive issues (e.g., accompanying polyneuropathy), balance organ disorders (e.g., bilateral vestibulopathy), and cognitive impairments (Lord et al., 2003; Ambrose et al., 2013; Hamacher et al., 2018). In this review, we focus on the impact of cognitive performance in gait abnormalities, which may contribute to pathophysiological mechanisms of FOG in PD patients (Walton et al., 2018).

Motor-cognitive dual-task studies have shown a reduction in gait speed, along with other gait performance parameters like variability, especially when attention sharing is required and prioritized between the two tasks (Ebersbach et al., 1995; Beauchet et al., 2005; Springer et al., 2006). This occurs already in mild to moderately affected PD patients (O’Shea et al., 2002). The secondary task may be cognitive (e.g., serial subtraction, digit span) (Hausdorff et al., 2003; Yogev et al., 2005; Plotnik et al., 2011a) or another motor task like hand movements; the type of secondary task had a mostly negligible effect on the performance decrement. Amplified gait variability can increase the chances of falls, negatively affecting quality of life and overall daily activities in PD patients (Bloem et al., 2004).

Turning the direction of gait is a complex task, requiring the integration of multiple sensory inputs, postural transitions, and inter-limb coordination. Therefore, it can be easily disturbed by challenges to attention and/or executive functions (Morris et al., 2016). Evaluation of the number of steps required for a full 180° turn while walking is part of the standardized clinical exam in PD, for which numerous online video resources exist.^1^

Overall, a wide array of cortical and subcortical regions is needed for goal-oriented gait and walking. “Executive function” describes higher-order sensory information processing within the frontal cortical and subcortical regions among their associated networks. It includes interpreting and analyzing task-related information content and modulating behavior by action and/or inhibition. The executive attention system has also been related to abnormal gait measurements (Morris et al., 2016). Anatomically, this includes the dorsolateral prefrontal cortex, anterior cingulate gyrus, posterior parietal, and the middle and superior temporal gyri with their mutual and thalamic interconnection (Gopher, 1996; Johansen-Berg and Matthews, 2002; Sheridan and Hausdorff, 2007). On a functional level, connectivity within the frontoparietal network has been linked to gait speed in older adults, whereas gait stride time variability has been linked to the strength of anti-phase functional connection between the dorsal attention network and the default network, which activates during the absence of cognitive challenge (Lo et al., 2017). Self-awareness, planning, response inhibition, and response monitoring needed for proper gait stability were functionally associated with thalamic and basal ganglia subcortical regions. These functional circuits can modulate lower-order automated locomotor circuits, which are more akin to those well-studied in decerebrated cats (Takakusaki, 2017).

The ability to manipulate and analyze spatial location and bearing is required during movement to avert falls by correct orientation and position. Morris and colleagues have found that slower steps and turning of gait are associated with poorer attention, as computed by using Stroope reading scale, and lack of visuospatial abilities linked with FOG (Morris et al., 2016). Domellof and colleagues assessed gait stability, as quantitative gait assessment of UPDRS subitems, and observed a consistent association with visuospatial abilities (Domellof et al., 2011). Our recent study using regression models suggests that FOG severity may be an indicator of a more global cognitive impairment, and that disease severity is correlated with both FOG and abnormal cognitive functioning scores (Scholl et al., 2021). Consequently, cognitive training has been shown to improve gait functions in PD patients with FOG (PDFOG+), compared to PD patients without FOG (PDFOG–) (Walton et al., 2018). Given the influence of cognitive domains on these motor dysfunctions, we suggest that gait abnormalities and FOG may occur by a simultaneous progression of motor disease severity and advancing cognitive impairment along the disease course of PD (Morris et al., 2020). However, more detailed dual-task studies need to illuminate the differential involvement of these two factors of PD gait disturbance. The current review aims to understand oscillatory activities in different frequency bands (theta band: 4–7 Hz, alpha band: 7–13 Hz, beta band: 13–30 Hz, and gamma band: 30–50 Hz) in the major cortical and subcortical regions in the motor-cognitive networks during gait and dual-task performances.

## Cortical oscillations during gait and dual-task

### Cortical theta band oscillations

Due to its flexibility and ease of application, scalp electroencephalography (EEG) provides ample studies to examine movement-related neural oscillations in PD and other diseases. Spectral power increases in the theta frequency band have been observed in the supplementary motor cortical and premotor cortical regions in young healthy subjects during walking with an obstacle task (Nordin et al., 2019). Additionally, synchronized theta band power in the left primary motor area in young healthy subjects has been correlated with the amount of acceleration of the contralateral right lower limb during the acceleration phase (Ofori et al., 2015). In conjunction, these studies underline the role of theta oscillations in lower-extremity motor performance of the respective contralateral limb.

According to current evidence, cognitive control for goal-directed movement is assumed to be mediated by midfrontal theta band activity in healthy and PD conditions (Cavanagh and Frank, 2014; Singh et al., 2018, 2021). Since FOG is related to attentional deficits, we see modulation in the theta band during both gait and cognition functions, demonstrating its potential role in dual-tasks. Decreased midfrontal theta activity was found during lower-limb pedaling initiation and execution in PDFOG+, but not among PDFOG– groups (Singh et al., 2020). By contrast, episodes of freezing were related to a significant increase in theta band power within the central and frontal cortical areas (Shine et al., 2014). These studies support the notion that an underlying mechanism for FOG can be differentiated from walking mechanisms in PD patients related to activity in the theta band. We observed similarity in theta band responses during cognitive function and lower limb movement activity. Interestingly, our previous study revealed that PD patients exhibited attenuated midfrontal theta activity related to response conflict and post-error processing during a Simon reaction time cognitive task (Singh et al., 2018). Even in rodents with disrupted cortical dopamine, researchers observed significantly reduced midfrontal theta activity during an interval timing cognitive task (Kim et al., 2017). Altogether, it appears that the cortical theta band network can play a crucial role in performing the gait task in PD. Higher activity in this cortical theta network appears to be observed, along with increased cognitive loading in high order dual-tasks.

It is notable that the effects of levodopa in the midfrontal theta dynamics were not found during cognitive and lower-limb motor tasks (Singh et al., 2018, 2020, 2021). This raises questions about the sensitivity of frontal cortical areas to dopamine and the need for other modes of treatment for clinical symptoms associated with this network. Moreover, deep brain stimulation (DBS) reveals mid-prefrontal cortical and basal ganglia coupling in theta band during high conflict cognitive processing suggesting the role of cortico-basal theta network in decision making (Zavala et al., 2016; Kelley et al., 2018; Cole et al., 2022). This appears to be essentially involved during gait and dual-task.

### Cortical alpha and beta bands oscillations

Most EEG studies have explored beta oscillations in the sensorimotor, motor cortex, and frontal regions (Pfurtscheller and Lopes da Silva, 1999; Brazhnik et al., 2012; Devergnas et al., 2014; Khanna and Carmena, 2017; Singh et al., 2020), whereas magnetoencephalography (MEG) recordings show both coherent alpha band activity in various temporal lobe locations and beta band activity in the sensorimotor and premotor cortical regions (Hirschmann et al., 2011). This study described the patterns of frequency-specific functional connectivity between basal ganglia and cortex, thus implying that inter-regional interactions may be isolated in the frequency domain. Interestingly, a normal gait with an oddball paradigm study observed reduced alpha oscillations in a static condition, with likely phenomena of desynchronization related to cognitive processing. In contrast, the alpha activity was canceled during gait, thus suggesting a condition of “idling” of cortical regions previously involved in the process of identification of the target stimulus (Tommaso et al., 2015). In a dual-task study, differences in spectral power at different bands except the alpha band were seen in PD patients compared to the single-task. In response to dual-task walking, power in the delta and theta bands was decreased in PD, whereas power in the beta band was higher compared to the healthy group (Possti et al., 2021). In a recent study, EEG collected during walking with visual cues in PD participants observed that brain activity differences with visual cues correlated with gait improvements, and PDFOG+ with cognitive deficits required more visual attentional processing accompanied by reduced alpha band activity in the occipital cortical region (Stuart et al., 2021). Other studies in PD have shown coherent activity in the alpha band between the temporoparietal-brainstem network and the STN; by contrast the beta band displayed higher coherence in the predominantly frontal network (Lalo et al., 2008; Litvak et al., 2011). These studies outlined that STN activity related to or perhaps driven by activity in the cortical region in both the alpha and beta frequency bands. Overall, the cortical structures and alpha band networks may be involved in attentional and motor planning, whereas the beta band connectivity may be involved with the execution of the motor processes required during gait or dual-task performances.

Beta oscillations are linked with motor control and increase in power can be seen during upper-limb bradykinesia and motor impairments in PD (Singh et al., 2013, 2018, 2020). These oscillations have mostly been suppressed during voluntary movements (Jenkinson and Brown, 2011). Motor-cortical oscillations in the beta band are up-regulated in both unmedicated and early stage medicated patients with PD compared to age-matched controls (Pollok et al., 2012). In the young healthy population, walking-while-talking exhibited increased theta and beta power in frontal and parietal regions, whereas walking-while-texting showed decreased beta power in the frontal-premotor and sensorimotor cortical regions (Pizzamiglio et al., 2017). Motor cortical beta band explicitly decreased during anticipatory processes and movement execution, whereas an increase in frontal beta band activity reflected cognitive top-down control, such as motor inhibition during step shortening in young healthy subjects (Wagner et al., 2016). While our previous report revealed increased midfrontal beta band power in PDFOG + compared to age-matched healthy controls during lower-limb motor initiation and execution with the attentional stimulus, no significant difference in the beta band power was seen in PDFOG + compared to PDFOG– (Singh et al., 2020). However, differences within these PD subgroups were prominent in the midfrontal theta frequency band at motor initiation. This study suggests that midfrontal beta power may not be specific to lower-limb motor abnormalities in PD patients. In another study, desynchronization of beta band was seen in the transition period before upper limb freezing, compared to conventional finger tapping in PD patients (Scholten et al., 2020), and the transition from walking to freezing was also associated with increased beta band activity in the parietal cortical region (Marquez et al., 2020), suggesting a distinct mechanism involved in upper limb freezing compared to lower limb freezing. Another instance of increased beta modulation was detected in the motor cortical region in healthy population, when the observed action was erroneous, thus supporting its cognitive characteristic (Koelewijn et al., 2008). Although levodopa treatment reduces pathological coherence in the beta band over the right frontal cortical region (Palmer et al., 2010; Singh, 2018), gait and dual-task performances were not necessarily improved by treatment, specifically along the progression of disease. The influence of beta band seems to vary in different brain regions, but it may be a contributing factor for achieving precise gait in PD.

### Cortical gamma band oscillations

Fewer studies have observed cortical gamma oscillations in association with gait in PD patients. Most gamma band studies were designed to investigate the response to the involuntary dyskinetic motor dysfunction. Swann and colleagues demonstrated that dyskinetic movements in PD subjects can be linked to narrow-band gamma oscillations in the motor cortex between 60 and 90 Hz (Swann et al., 2016). However, an electrocorticographic study in clinical subjects found event-related synchronized gamma oscillations with somatotopic organization over the sensorimotor areas, which tended to be synchronized to the onset of a movement; this stood in contrast to lower frequency beta band oscillations in healthy participants (Crone et al., 1998). In a more recent study, the respective peak frequency of gamma activity was significantly lower during foot dorsiflexion than during finger abduction and elbow flexion in healthy participants (Cheyne et al., 2008). Interestingly, increased gamma oscillations have been noticed primarily over central sensorimotor areas in young healthy subjects during walking compared to standing (Seeber et al., 2015), and central-parietal cortical areas during challenging postural tasks in elderly people, indicating increased distribution of attentional sources to postural tasks (Ozdemir et al., 2016). A recent rodent study observed that rapid decreases in motor cortical gamma field potential activity contributed to evolving plasticity supporting higher beta range synchronized activity in basal ganglia-thalamocortical circuits after a loss of dopamine receptor stimulation (Brazhnik et al., 2021). Moreover, high beta-gamma phase-amplitude coupling in the primary motor cortical region was found in freezing episodes only. This high coupling in freezing episodes was not caused by dual-tasking in PD patients, suggesting the role of increased phase-amplitude coupling in the primary motor cortex only in the occurrence of a freezing phenomenon (Yin et al., 2022). Altogether, these studies show that cortical gamma oscillations are likely to modulate during interference and may play a crucial role in dual-task performances.

## Subcortical oscillations during gait and dual-task

Functional neurosurgery using DBS has proven to be an effective therapeutic treatment for individuals with PD and other neurological disorders. To better comprehend electrophysiological activity, DBS leads are inserted at various depths in the subcortical structures. Though quite invasive, DBS surgery serves as a unique opportunity to record local field potential (LFP) activity in specific areas of the basal ganglia in humans. Other areas are only accessible in animal studies with limited and less predictable applicability for human diseases. The LFP signal is an extracellular field generated primarily by synaptic transmembrane currents recorded from a cluster of neurons surrounding the depth DBS electrode (Yin et al., 2021). It reflects the synchronous activity of several hundreds to thousands of neurons in the immediate vicinity of the lead, in contrast to single-cell recordings. In LFP the proportional intensity or silencing of frequency bands during experimental conditions can be recorded, but not individual cell activity or the proportion of projection neurons versus local interneurons. The therapeutic DBS approach gives researchers an advantage to explore subcortical basal ganglia oscillations either via externalizing DBS leads (Singh et al., 2011a,^2013^) or a wireless system using the Medtronic Percept™ (Koeglsperger et al., 2021). Neuroimaging studies have shown the functional connectivity in the cortical and subcortical networks, however, to understand the extensive pathophysiology of the subcortical regions, LFP signals offer a direct insight into parts of the basal ganglia function in PD (Brown, 2003). Much of the current understanding of PD pathophysiology has been defined by the abnormal neuronal activity of the basal ganglia direct and indirect pathways (Singh, 2018). Abnormal neuronal activity in PD includes increased burst discharges, oscillatory firing, and synchronous firing patterns throughout several instances of the basal ganglia regions (Rubin et al., 2012).

The term basal ganglia comprise structurally and functionally interconnected input/output and intrinsic nuclei. Input striatal nuclei like the caudate nucleus, putamen, and accumbens nucleus receive information from the cortical regions, which are then processed and relayed to the output nuclei, consisting of the GPi and SNr. Both output and input nuclei are connected through the intrinsic nuclei, like the globus pallidus externus (GPe), STN, and substantia nigra compacta. The extent to which information is propagated along the basal ganglia network in PD compared to the healthy condition on different scales is still under debate, from a single-cell based relay with diverse types of neurons, long-term chemical potential storage, up to the population scale neural oscillatory band activity (Blenkinsop et al., 2017; Martin et al., 2019). Each of these levels have today been established as separate research fields.

Several studies have recently illuminated new aspects of oscillatory activity propagation. Striatal cholinergic interneurons induced beta and gamma oscillations in cortical-striatal circuits and were able to influence motor behavior in mouse models (Kondabolu et al., 2016). This study also proposed that striatal cholinergic interneurons may mediate the emergence of abnormal exaggerated beta oscillations within cortico-basal ganglia thalamic networks of normal mice and induce parkinsonian-like motor deficits. Most of the LFP recordings during upper- and lower-limb motor and cognitive functions from the human basal ganglia have been studied in the STN and the GPi, which are therapeutic targets for DBS (Bergman et al., 1990). In this review, we focus on these regions.

The synergistic interaction between the STN and GPi has been known to affect gait (Singh et al., 2011a,^2013^; Singh, 2018), since increased STN firing is expected to enhance GPi activity, resulting in thalamic disfacilitation of the cortical region and, consequently, inhibition of motor performance in PD (Frank, 2006; Bogacz and Gurney, 2007; Zavala et al., 2015). Interestingly, afferent and efferent projection to the basal ganglia also derives from the pedunculopontine nucleus (PPN) (Mena-Segovia et al., 2004). PPN is thought to be involved in the initiation and modulation of gait, which has been demonstrated when the electrical stimulation and application of neuroactive substances in the PPN elicited locomotor activity in monkeys (Pahapill and Lozano, 2000). Overall LFPs from these subcortical areas provide valuable insight on the nature of the oscillations during gait and more complex situations like dual-task.

### Oscillations in the subthalamic nucleus

#### Subthalamic nucleus theta band oscillations

Subcortical theta and alpha oscillations in PD are often described together, since most studies have referred to both as “low- frequency” for their analysis (Giannicola et al., 2013; Wojtecki et al., 2017). While many oscillatory studies in the STN focused mostly on beta frequency in PD, Giannicola and colleagues suggested that the low frequency bands can be used as a control variable in adaptive DBS systems (Giannicola et al., 2013). Consequently, theta band modulation has been discovered to serve as a spectral correlation for FOG, with activity focused on the lower-most contact pair of DBS lead implanted into the motor portion of the STN (Chen et al., 2019). Low-frequency baseline power was also seen to be comparable with and without levodopa. However, DBS with levodopa pre-treatment increased low-frequency oscillations (Rossi et al., 2008). It is unclear how this may affect gait or dual-task in PDFOG+, therefore additional investigation is required. Like cortical theta networks, the STN theta network activity has been seen in PD patients during cognitive tasks, and its association with the prefrontal cortical region has been demonstrated (Kelley et al., 2018; Cole et al., 2022; Navid et al., 2022). Interestingly, simultaneous STN LFPs and scalp EEG recordings during walking displayed a synchronization in a low frequency band (4–13 Hz); however, no differences in STN theta or interhemispheric STN coupling were observed during FOG episodes when compared to effective walking (Pozzi et al., 2019). Another study observed increased theta power in the STN LFPs during periods of vulnerable gait in both single-task and dual-task gait in PD patients (Chen et al., 2019). In a recent rodent study, electrophysiological recordings with optogenetic manipulations of projections from the mid-prefrontal area to STN found that gamma oscillations were coordinated between the prefrontal area and STN at the theta band during a cognitive-motor task (Heikenfeld et al., 2020). This study suggests that action selection in a cognitively demanding task (gait or dual-task) may involve theta frequencies coordination of higher oscillations signaling in the prefrontal-subthalamic hyperdirect pathway. Altogether, abnormal gait in PD can be characterized by cortical-subthalamic decoupling in the theta network at the transition from normal walking into FOG events or gait with cognitive loading. These reports propose future correlational studies between STN theta network, influence of cognitive load, and single- and dual-gait motor tasks.

#### Subthalamic nucleus alpha band oscillations

Subthalamic nucleus LFPs recorded from an implanted sensing neurostimulator (Medtronic Activa PC + S) during walking in PD patients showed a modulation in alpha frequency power, which was locked to the gait cycle (Hell et al., 2018). These oscillations may represent physiological modulation during gait, since similar changes have been observed in dystonia patients without gait deficits (Singh et al., 2011a). Increased power in the alpha band has also been observed in the STN LFPs in PD patients during a ballistic upper-limb motor task (Singh et al., 2011b). STN LFPs during a stepping task revealed differences in alpha frequency entropy between PDFOG + and PDFOG– groups, with a higher alpha entropy in PDFOG + during FOG episodes (Syrkin-Nikolau et al., 2017). This study suggested that conversely increased entropy in alpha/beta oscillations may be a compensatory effort to improve the gait and dual-task problems in PDFOG +. There was no difference between effective walking compared to FOG episodes in both STN theta and alpha power; yet alpha power was higher during normal walking in the cortical-STN networks (Pozzi et al., 2019). A recent clinical DBS study demonstrated the superiority of combined DBS of STN and substantia nigra in improving temporal stepping variability of the affected leg in PD patients compared to conventional STN DBS (Wagner et al., 2022). This study also demonstrated that active DBS reduced cortical alpha power during stepping movements in PD patients. During STN DBS only, FOG episodes were associated with an increase in cortical alpha and low-beta oscillations. Furthermore, it has been described that STN alpha band power associates with frontal and premotor cortical areas (Horn et al., 2017), which are linked to attention and executive motor functions, especially those that are technically challenging such as gait-cognitive dual-task (Jahanshahi, 2013). For example, during a turning and barrier course task that requires cognitive challenge, an increase in alpha entropy was seen during gait with FOG (Syrkin-Nikolau et al., 2017). Altogether, studies of STN alpha band oscillations in conjugation with cortical regions demonstrate that these oscillations may reflect impaired information processing in the attention-related cortico-STN hyperdirect pathway. This might however, also be part of a compensatory mechanism to abnormal gait and dual-task in PD.

#### Subthalamic nucleus beta band oscillations

Unlike the low-frequency oscillations, STN beta band oscillations have generally been examined specifically in response to gait in PD. A recent study demonstrated gait-related peak frequency modulation in the STN beta band in PD patients and classified beta-related neural features, which could consistently differentiate between the standing and walking conditions in each PD patient (Canessa et al., 2020). An increase in beta band oscillations was highly correlated with abnormal gait pattern in PDFOG + and might possibly represent a key component in the causal mechanism for severe akinesia during gait in PD, since PDFOG + exhibited enhanced low beta frequency power during walking when compared to PDFOG– (Singh et al., 2013). These outcomes were supported by Toledo et al. (2014), who reported higher power in the beta band for PDFOG + versus PDFOG–, along with a reduction in beta power after levodopa administration in conjunction with a suppression of FOG events. These studies with increased beta in lower-limb motor activity were altogether opposed to numerous upper-limb motor studies, which showed a suppression of akinetic beta oscillation in ongoing motor reaction time performances (Kuhn et al., 2004; Williams et al., 2005), suggesting a differential beta oscillatory use in the basal ganglia for upper versus lower limb motor performances. The effects of levodopa on beta band oscillations have been demonstrated with significantly positive correlation between a decrease in the 8–35 Hz power and an improvement in upper limb motor function, when measured by motor assessment scores (Kuhn et al., 2006). Several studies have highlighted suppression in beta activity in upper-limb motor execution, as well as in motor preparation (Kuhn et al., 2004; Litvak et al., 2011; Canessa et al., 2020). This was in contrast to lower limb movement, which was not equally responsive to levodopa treatment in PD with severe lower extremity abnormalities. Additionally, dopamine only modulated low frequency oscillations, since participants in the “ON” state after levodopa administration displayed a profound reduction in exclusively low beta activity compared to high beta activity (Brown et al., 2001; Singh et al., 2013).

The implication of movement preparation on the beta band illustrates the role of cognitive control. Does the planning process quantitatively influence the low frequency oscillations as the motor execution did, and can an improvement in cognitive function modulate the beta band oscillations for execution? This may prove to play a crucial role in cognitive control and improved gait stability. A model has been proposed in which successful action selection and decision making relies on an intact STN activity, which regulates the temporal dynamics of motor control during conflict and inhibits movement until sufficient information to select the right option has been integrated (Frank, 2006; Bogacz and Gurney, 2007). So far, few studies have shown a synergistic interaction between cognition and STN models. Chen and colleagues found that PDFOG + had significantly higher relative power in the beta and theta bands during periods of vulnerable gait in both single and dual-task states, and a narrow band around 18 Hz may be linked to FOG events (Chen et al., 2019). Interestingly, STN LFPs from an implanted Activa^®^ PC + S sensing neurostimulator during gait demonstrated sustained beta burst durations in PDFOG + compared to PDFOG– as a pathological neural feature of FOG. The beta burst durations were shortened during DBS along with an improvement in gait kinematics (Anidi et al., 2018). Another study used the same DBS device and reported improvement in gait outcomes with DBS and showed a decrease in high beta frequency power (20–30Hz) with bilateral oscillatory connectivity during gait (Hell et al., 2018). A reduction in overall high beta patterns, such as burst amplitude and burst duration, was observed during gait when compared to resting OFF DBS. This study argued that the observed oscillatory changes might be related to movement induced artifacts or reafference, which may have important implications for gait-related research (Hell et al., 2018). Later, the same group recorded STN LFPs using the Medtronic Percept™ neurostimulator during gait in response to DBS, and found a double-peaked beta activity, which decreased with increasing stimulation intensity and gait activity (Koeglsperger et al., 2021). In this study, a new peak activity at 13 Hz was established. In support to these human studies, hemiparkinsonian rodent studies found an increase of beta power during walking in the substantia nigra pars reticulata and the STN (Avila et al., 2010; Delaville et al., 2015). This modulation was correlated to the kinematics of contralateral paw movement. One hypothesis originating from these studies was that a lack of dynamic modulation in the STN beta band oscillations may compromise cortico-basal ganglia processing related to gait and dual-task control in PD. Altogether, it has been proposed that the STN beta band power modulations may detect gait abnormalities in PD patients for future use as a feedback parameter in closed-loop DBS neurostimulation.

### Subthalamic nucleus gamma band oscillations

Another frequency band prominent in the cortical-basal ganglia loop in PD lies within the gamma frequency band. Most movement-related studies on the gamma band oscillations focused on upper limb movement in PD. Impairment in grip force activity was correlated with lower gamma activity in the STN (Tan et al., 2013). However, both PDFOG + and PDFOG– displayed an increase of STN gamma oscillations (60–90 Hz) during walking (Singh et al., 2013), showing that lower limb activity may have a different mechanism in response to gamma activity. A study that combined STN LFP and MEG found increased motor cortical-STN coherence in the gamma band during synchronous and sequential finger movements, indicating that gamma oscillations were prokinetic, but likely through a modulatory effect (Litvak et al., 2012). Numerous studies have shown a correlation between an increase in gamma activity and improvements in motor impairment upon dopaminergic treatment (Kuhn et al., 2006; Litvak et al., 2011; Elben et al., 2018; Lofredi et al., 2018). STN LFPs during gait cycles exhibited modulation in gamma oscillations along with other frequency oscillations during gait in PD (Hell et al., 2018). We therefore suggest that STN gamma activity can be relevant for gait-related oscillation studies and could be used to understand its response to dopamine in coherence with the motor cortical region.

### Oscillations in the globus pallidus internus

Previous studies have analyzed both STN and GPi structures simultaneously, revealing similar effects and suggesting that both basal ganglia nuclei can share similar oscillatory patterns in PD patients (Gatev et al., 2006; Nambu and Tachibana, 2014). One possible reason for that would be because the GPi serves as an output structure for the STN with synergistic interactions and synchronization. Brown and colleagues have shown that both low and high frequency STN stimulation can normalize the abnormal oscillatory pattern of the basal ganglia output structures, including GPi (Brown et al., 2004). Understanding the possible coupling between STN and GPi structures may help increase post-synaptic efficacy in advanced therapeutic neuromodulation approaches. Synchronization in the human subthalamo-pallidal circuit occurs at a variety of frequencies, depending on the degree of dopaminergic activity present (Brown et al., 2001). LFP recordings from GPi and STN in PD patients with off levodopa demonstrated that activity in GPi could control activity in STN at ∼20 Hz (Brown et al., 2001). In untreated PD patients, STN activity at 6 Hz outpaced corresponding activity in GPi, suggesting that output from GPi was synchronized at low frequency oscillations (Brown and Marsden, 1998). However, Levy et al. (2000) suggested that the frequency of synchronization may not be the frequency of discharge of individual neurons even within LFP, but instead it represents a population effect, in which some of the individual single-cell discharges occur at the same frequency, which may or may not be higher than the discharge rate of individual units. These oscillatory activities appear to be modulated by the input basal ganglia structures. Distinct features of the output GPi oscillations were found in recent studies. While elevated beta frequency band played a role in PD, exaggerated movement-modulated high-frequency oscillations in the GPi might also be pathophysiological features of PD (Johnson et al., 2021). This study also identified a profound oscillatory peak at 200–300 Hz, which was elevated during movements occurring in negative correlation with other bradykinetic movements. By contrast, PD with pronounced rigidity and bradykinetic features showed predominately high beta oscillatory power in the GPi, whereas subjects with more pronounced FOG and gait problems displayed a broader oscillatory spectrum with a prominent double peak at low and high beta frequency bands (Aman et al., 2020). An increase in normal gait speed can alter the power of GPi oscillations with a reduction of the activity in the low beta band and an upregulation of activity in the gamma band (Singh et al., 2011a,^2013^). Overall, the role of GPi oscillations in the cognitive control is still unclear, as is the role of the STN in action selection. A recent study demonstrated GPi theta activity during cognitive processing and proposed that the GPi may be necessary for cognitive processing, similar to the STN (Navid et al., 2022). It is still unclear whether specific GPi oscillations could be responsible for action selection and kinematic features of FOG in PD. A robust lateralized functional connectivity between the globus pallidus and motor cortical region was found among PDFOG + patients, suggesting a strong predictor of FOG severity (Miranda-Dominguez et al., 2020). Interestingly, a recent study demonstrated the correlation between the effects of GPi DBS on FOG and disease duration, suggesting the useful information to predict improvement in FOG after GPi DBS in PD patients (Lee et al., 2022). Since GPi is not a common target for therapeutic stimulation in PD, there is a gap in understanding the contribution of GPi oscillations in abnormal gait and dual-task in PD patients. Therefore, more GPi recordings are warranted during lower-limb motor and cognitive tasks to understand their deeper role in PD symptomology and possible interventions for clinical benefit.

### Oscillations in the pedunculopontine nucleus

Studies have shown that gait and dual-task interferences are highly correlated with an asymmetry in PPN structural and functional connectivity, applied during both simple and complex cognitive tasks in PDFOG + versus PDFOG– (Pahapill and Lozano, 2000; Thevathasan et al., 2012; Peterson et al., 2015; King et al., 2020). DBS of the PPN has been postulated as a potential therapy for PD patients with pronounced gait issues (Ferraye et al., 2010; Moro et al., 2010). Due to its broad reciprocal connections with basal ganglia nuclei, thalamus, reticular formation, deep cerebellar nuclei, and the spinal cord, the PPN functions as a pivotal region for locomotive activities (Pienaar et al., 2017). PPN alpha band oscillations are most prominently prevalent in gait and dual-task in PD patients. This finding has linked PPN to the gait performance in PD patients (Thevathasan et al., 2012; Tattersall et al., 2014). Gait freezing is associated with decreased alpha band oscillations, however, beta peaks are less consistently observed during gait tasks (Thevathasan et al., 2012). Levodopa can enhance PPN alpha band oscillatory synchronization and bidirectional coupling with the cortical EEG, underlining a likely role in PD gait pathology (Androulidakis et al., 2008; Fraix et al., 2013). Altogether, these studies suggest that the coupling between PPN oscillations to cortical alpha activity is a functional correlate of motor attentional processes required to perform challenging gait and dual-tasks. Although prominent in lower limb tasks, alpha band activity was not found during self-paced wrist and ankle movement, suggesting its distinct role in locomotion-specific lower limb control (He et al., 2021). There are indications of association with attentional control mechanisms. In a study where participants’ reaction times were compared before and after bilateral DBS, low-frequency stimulation of PPN improved basal attentional processing with differences observed in their reaction time (Fischer et al., 2015). Bilateral stimulation of the mid-caudal PPN proved helpful for PDFOG + with postural instability and frequent falls, increasing local alpha activity (Thevathasan et al., 2011). Ferraye et al. (2010) also observed general gait improvement in UPDRS for some PDOFG + during ON versus OFF DBS states, although some participants did not show any improvement. These studies suggested that PPN alpha band oscillations could serve as an important measurement parameter or even mode of influence for attentional control impacting PD gait performance.

Another recent study demonstrated a correlation between contralateral foot gait phase and PPN beta band modulation in PD, which was most pronounced during regular stepping rhythms (He et al., 2021). Fraix and fellows observed increased PPN alpha oscillations during a step-in-place task along with decreased beta oscillations ON levodopa, suggesting that the gait difficulties in PD could be related to an imbalance between low and higher PPN frequencies (Fraix et al., 2013). PPN theta band oscillations have not been thoroughly investigated during either lower and upper limb movements. A single study showed greater theta event-related desynchronization and beta event-related synchronization with upper-limb movements ON levodopa, suggesting that beta oscillations may have differential functions in the basal ganglia and PPN regions (Tsang et al., 2010). Interestingly, a reciporcal downstream effect has been suggested in the 6-OHDA lesioned rat, wherein stimulation in the PPN reduced beta activity in the STN (Alam et al., 2012). Particularly here, further experimentation is required to understand this reciporcity and its effect between both nuclei and the wider framework.

Moreover, during the ON phase, there was a decrease in PPN gamma band oscillations for PDFOG +; this activity was only seen during gait and not in sitting and standing (Fraix et al., 2013). Modulations in PPN gamma band oscillations under levodopa could be related to an increased alertness. Therefore, one prominent theory that summarizes the functional roles of the PPN gamma and alpha bands oscillations requires cognitive control in synergy with locomotion in order to achieve gait stability specifically during a dual-task. Overall, profound PPN alpha band oscillatory activity may regulate attentional processes to improve gait and dual-task performance in PD. PPN DBS can alleviate gait abnormalities by driving PPN neurons and thereby normalizing alpha oscillations. Overall, PPN-DBS treatment remains experimental and only a small experimental PD group is available to explore neural activity in the PPN and connected networks.

### Oscillations in the cerebellar region

Cerebellar regions exhibit functional connectivity with extensive motor- and cognitive-related regions. Not much attention has been given to the cerebellar gait-related oscillations, even though activation in the cerebellar region has been observed during both actual and imaginary walking (Fukuyama et al., 1997; Jahn et al., 2008). In a study among patients with ischemic stroke, cerebellar magnetic stimulation coupled with physiotherapy could improve gait and balance functions by supposedly enhanced cerebello-cortical plasticity (Koch et al., 2019). Some PD studies showed increased activation in the cerebellum during performance of various upper limb movements (Lewis et al., 2013; Wu and Hallett, 2013), which has been suggested to serve as a compensatory effect increasing with cerebellar disease severity (Sen et al., 2010; Muller et al., 2013; Takakusaki, 2017). Modulation of the cerebellar LFP in the 13–25 Hz band with the elbow flexion-extension task have been found in normal monkeys (Courtemanche et al., 2002). Recent cerebellar EEG study observed increased theta-band oscillations in PD patients compared to age-matched healthy controls, suggesting the pathophysiological role of cerebellar oscillations in PD (Bosch et al., 2021b). The same research group further studied cerebellar oscillations during lower-limb movements and temporal cognitive processing tasks in PD patients, demonstrating attenuated cue-triggered theta-band power over a mid-cerebellar electrode during both tasks (Bosch et al., 2021a). The cue-triggered mid-cerebellar theta activity in the lower-limb pedaling motor task was also correlated with gait and cognitive impairments in PD (Bosch et al., 2021a). Similar results have been discussed by De Zeeuw and colleagues, where cerebellar cortical theta band oscillations (4–9 Hz) were attributed to learning-dependent timing (De Zeeuw et al., 2008). Additionally, another EEG study showed profoundly lower power in theta band in both mid-cerebellar and mid-frontal regions during the postural control task in PD patients with postural instability when compared to PD patients without postural instability and healthy control groups (Bosch et al., 2021c). Moreover, cerebellar beta oscillations showed no association with gait impairment in PD (Bosch et al., 2021a). A dual-task functional MRI study demonstrated activation in the right cerebellar vermis and left lobule V of the cerebellar anterior lobe, illustrating that some cerebellar regions can be additionally activated with dual-task performance (Wu et al., 2013).

Cerebellar gamma oscillations have not been fully investigated during gait. Intracranial EEG of the human cerebellar cortex may exhibit electrical rhythms as high as 250 Hz, including task-related modulations and these oscillations are in line with electrophysiological studies of cerebellar oscillations in the rodent as well as magnetoencephalographic findings in humans (Dalal et al., 2013). It should be noted that the unique cerebellar histological architecture is widely different from the six-layered neocortex regions, and therefore frequency bands in cerebellar EEG are also different (Andersen et al., 2020; Bosch et al., 2021c). Further investigations are required in the cerebellum region due to it prominent locomotive nature.

## Conclusion

We have summarized previous studies on cortical and subcortical oscillations during gait and dual-tasks in PD in Table 1. The oscillatory patterns of cortical and subcortical motor-cognitive systems have seen extensive individual studies on their correlation with gait disturbance in PD. Few characteristic frequency bands have been identified as measurable markers, possible outcome variables, or even promising therapeutic targets to alleviate PD gait instability, particularly FOG. Numerous studies focusing on the cortical and sub-cortical regions in response to gait mechanism may be greatly simplifying the integration of numerous systems involved. Our review examines the comprehensive role of the main gait and dual-task networks in PD. Notably, many researchers studied cortical oscillations during gait and dual-task in young heathy subjects, however, the PD population is relatively older, therefore, conclusions related to the oscillatory nature during these motor tasks cannot be considered entirely comparable between these groups. There is no sufficient data on how age alone as a factor influences LFPs in human subjects, especially among the subcortical structures with the additional presence of motor disorder.

**TABLE 1:** Summary of the studied oscillations during gait/walking and dual-tasks in the cortical and subcortical structures in patients with PD.

Brain region	Oscillations	Motor task	References
Cortical region (EEG/ECoG)	Theta-band	Walking	Ofori et al., 2015
		Walking with obstacle	Nordin et al., 2019
		Pedaling task	Singh et al., 2020
	Alpha-band	Gait	Tommaso et al., 2015
		Walking with visual cues	Stuart et al., 2021
	Beta-band	Auditory oddball with walking	Possti et al., 2021
		Pedaling task	Singh et al., 2020
		Walking while talking/texting	Pizzamiglio et al., 2017
		Step shortening	Wagner et al., 2016
		Walking while finger- tapping	Scholten et al., 2020
	Gamma-band	Walking	Swann et al., 2016
		Foot dorsiflexion	Crone et al., 1998
		Walking	Seeber et al., 2015
		Posture-cognition task (encoding and retrieval)	Ozdemir et al., 2016
Subthalamic nucleus (LFPs)	Theta-band	Walking	Chen et al., 2019; Pozzi et al., 2019
	Alpha-band	Walking/Gait	Hell et al., 2018; Pozzi et al., 2019
		Stepping task	Syrkin-Nikolau et al., 2017
		Stepping in place task	Wagner et al., 2022
	Beta-band	Standing vs. Walking	Canessa et al., 2020
		Walking	Singh et al., 2013; Toledo et al., 2014
		Walking and dual-gait task	Chen et al., 2019
		Gait	Hell et al., 2018
		Walking in rodents	Avila et al., 2010; Delaville et al., 2015
	Gamma-band	Walking/Gait	Singh et al., 2013; Hell et al., 2018
Globus pallidus internus (LFPs)	Beta-band	Upper-limb movement	Aman et al., 2020; Johnson et al., 2021
Pedunculopontine (LFPs)	Alpha-band	Gait	Thevathasan et al., 2012; Tattersall et al., 2014
		Stance and Stepping	Fraix et al., 2013
		Stepping in place or free walking	He et al., 2021
	Beta-band	Regular stepping	He et al., 2021
	Gamma-band	Gait	Fraix et al., 2013
Cerebellum (EEG)	Theta-band	Pedaling task	Bosch et al., 2021a
	Alpha-band	Postural control	Bosch et al., 2021c
	Beta-band	Pedaling task	Bosch et al., 2021a

The description of mere increases and decreases of frequency band relative power in relation to specific tasks or conditions do not constitute a causation or even necessarily a correlation, which has been criticized as a common fallacy in the field of LFP (Herreras, 2016). Also, the difference between extracranial cortical EEG recordings to truly local LFPs from intracerebral leads must be considered. Frequency bands are neither necessarily locally limited, nor do they reflect a specific action within the recorded nucleus. What may however, be considered helpful in the deeper understanding of the underlying mechanisms are specifically timed events within cyclic activity like gait, in conjunction with the respective disease or therapy conditions. In particular, studies with simultaneous recordings in mutually interconnected loci, combination with local oligo-cell recordings and e.g., optogenetic interference in animal models provide promising outlooks. The reviewed studies suggest that the theta and beta oscillations in the premotor cortical areas and basal ganglia, as well as alpha oscillations in the PPN, present specific modulation in response to clinically effective medical levodopa and direct electrical DBS therapies, in order to improve gait and dual-task performance in PDFOG+ compared to PDFOG– or healthy control groups. Based on these findings, specific approaches to location, modulation, and timing of therapies can be formulated for specific functional deficits, e.g., FOG, in the general degenerative condition of PD.

We emphasize that the cognitive network should be subject to further examination for improving overall gait dysfunctions in PD along the progression of the disease. Future studies, particularly those using mobile recording systems with high temporal and spatial resolution, like mobile MEG systems, will likely provide rapid and deeper insight into cortical and subcortical oscillations during gait-like leg movements and dual-tasks (Boto et al., 2018).

The development of new experimental techniques like high-density mobile EEG (Wagner et al., 2019) and wireless EEG (Maidan et al., 2019) now allow us to collect brain signals with high signal-to-noise ratio during gait and FOG episodes. Besides collecting subcortical LFPs via externalizing DBS leads, recently available Percept™ DBS devices from Medtronic company can be used to collect continuous LFP signals wirelessly during gait, and specifically during FOG episodes in PD patients. This is the first in probably a series of competitively available systems for treatment and study of intracerebral conditions among movement disorders, which may hopefully advance pathophysiological understanding. A previous study collected LFPs from the STN via the Percept™ DBS device in PD patients during gait and observed different peaks in the beta band, suggesting their relevance and usefulness as closed-loop biomarkers (Koeglsperger et al., 2021). Interestingly, the Percept™ DBS device performs closed loop stimulation with BrainSense™ technology and LFP collection at the same time. It is likely we will see new systems like these allowing us to explore the causal oscillatory mechanisms in the cortical and subcortical networks associated with gait dysfunction or FOG in PD patients in much greater detail and more refined experimental conditions in the future.

Long-term electrophysiological recordings starting in patients with the stage of PDFOG– and re-testing them along their individual progression to PDFOG + with gait and dual-task studies may further define the evolution of abnormal neural oscillations in the cortical and sub-cortical regions and networks. Evolving knowledge on these oscillations in health and disease, along with advances in neuromodulation therapies, particularly closed-loop DBS (Su et al., 2019), will help to alleviate gait dysfunctions in PD until, hopefully, the underlying molecular causes of PD synuclein neurodegeneration can be addressed with disease-modifying therapies before further disease progression can occur (Iarkov et al., 2020; Stoker and Barker, 2020).

## Author contributions

All authors listed have made a substantial, direct, and intellectual contribution to the work, and approved it for publication.

## Footnotes

1
https://stanfordmedicine25.stanford.edu/the25/parkinsondisease.html
